# Cholera-toxin-induced disease generates epithelial-cell-derived L-lactate that promotes *Vibrio cholerae* growth in the small intestine

**DOI:** 10.1016/j.celrep.2026.117509

**Published:** 2026-06-08

**Authors:** Maria de la Paz Gutierrez, Jenna L. Hall, Hugo L.P. Masson, Maria G. Winter, Sebastian E. Winter, Fabian Rivera-Chávez

**Affiliations:** 1 Department of Pediatrics, Division of Host-Microbe Systems and Therapeutics, School of Medicine, University of California, San Diego, 9500 Gilman Dr, La Jolla, CA 92093, USA; 2 Department of Molecular Biology, School of Biological Sciences, University of California, San Diego, 9500 Gilman Dr, La Jolla, CA 92093, USA; 3 Department of Medical Microbiology and Immunology, School of Medicine, University of California, at Davis, Davis, CA 95616, USA; 4 Division of Infectious Diseases, School of Medicine, University of California, at Davis, One Shields Ave, Davis CA 95616, USA; 5 Lead contact

## Abstract

Cholera toxin (CT) promotes *Vibrio cholerae* colonization by altering gut metabolism to favor pathogen growth. Here, we show that CT-induced disease leads to the upregulation of mammalian lactate dehydrogenase A (LDHA), an enzyme that catalyzes the conversion of pyruvate to L-lactate, in small intestinal epithelial cells. In a suckling mouse model, the bacterial L-lactate dehydrogenase (LldD) confers a fitness advantage to *V*. *cholerae* but not to the Δ*ctxAB* mutant incapable of producing CT. Finally, mice lacking epithelial-cell-specific LDHA have reduced luminal L-lactate concentrations, and the fitness advantage conferred by LldD is significantly reduced in these mice, demonstrating that epithelial-derived L-lactate is a major contributor to CT-dependent pathogen expansion. These findings identify epithelial-derived L-lactate as a host-derived metabolite generated in the small intestine during cholera disease that directly fuels *V*. *cholerae* growth during infection, uncovering a mechanism by which CT confers a fitness advantage to the pathogen during disease.

## In brief

Gutierrez et al. show that cholera toxin induces epithelial LDHA-dependent L-lactate production in the small intestine, creating a nutrient niche that *Vibrio cholerae* exploits through LldD-mediated L-lactate utilization to gain a fitness advantage during infection.

## Graphical Abstract

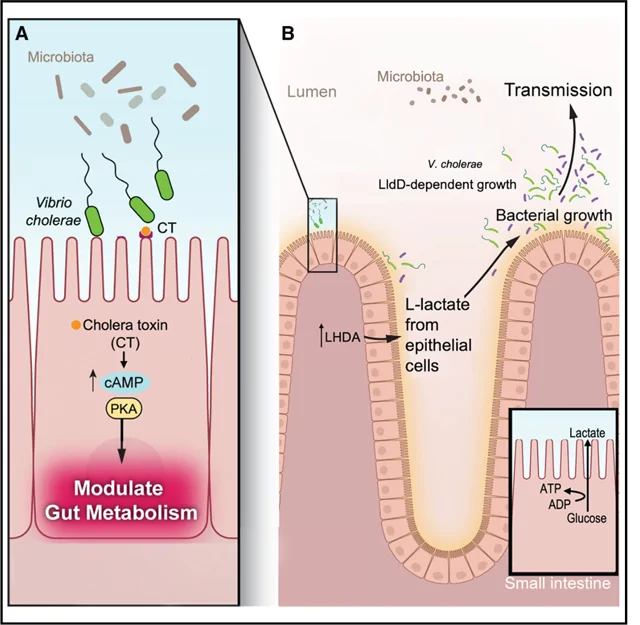

## INTRODUCTION

*Vibrio cholerae* is the causative agent of cholera, a severe secretory diarrheal disease that remains endemic in many parts of the world. Following ingestion, *V. cholerae* colonizes the small intestine, where it produces cholera toxin (CT), a potent enterotoxin that activates host adenylate cyclase and drives fluid secretion into the intestinal lumen.^1^ This secretory response results in the profuse watery diarrhea known as “rice water stool,” which underlies both disease symptoms and fecal-oral transmission of the pathogen.

In addition to promoting fluid loss, CT alters the metabolic landscape of the gastrointestinal tract. During infection, CT-induced disease drives marked changes in the composition of host-derived metabolites in the intestinal lumen of infant rabbits, including elevated levels of long-chain fatty acids (LCFAs), heme, and L-lactate. These changes coincide with a transcriptional program in *V. cholerae* enriched for genes involved in the tricarboxylic acid (TCA) cycle, iron acquisition, fatty acid metabolism, and L-lactate metabolism.^2^ The pathogen’s acquisition of both LCFAs and heme has been shown to promote growth during CT-induced disease.

Together, these findings suggest that CT not only drives diarrheal symptoms but also establishes a nutrient-rich environment that favors pathogen expansion. However, the mechanisms by which CT-dependent metabolic remodeling confers a fitness advantage to *V. cholerae* remain poorly understood.

Lactate is a glycolytic by-product produced under both aerobic and hypoxic conditions. It exists as two stereoisomers, D- and L-lactate, each metabolized by distinct, stereospecific dehydrogenases. While members of the gut microbiota can produce both isomers, mammalian tissues generate only L-lactate, primarily via the activity of LDHA. Several bacterial pathogens, including *Salmonella* Typhimurium and *Campylobacter jejuni,* use host-derived L-lactate during colonization of cecum and colon, respectively.^3,4^ Notably, epithelial-derived L-lactate has been shown to support the bloom of *Enterobacteriaceae* in the lumen of the large intestine.^5^ Although increased levels of L-lactate have been observed in the small intestinal lumen during CT-induced disease, the source of this metabolite remains undefined. Moreover, it is unknown whether L-lactate contributes to *V. cholerae* growth in the small intestine.

*V. cholerae* encodes a putative L-lactate dehydrogenase (LldD) and an L-lactate permease (LldP), both of which are transcriptionally upregulated in a CT-dependent manner during infection of the terminal ileum of the small intestine.^2^ However, whether *V. cholerae* actively uses L-lactate *in vivo* remains unknown. In parallel, the contribution of CT-induced changes in host epithelial metabolism to *V. cholerae* colonization is also not well understood.

Here, we investigated whether CT-induced disease promotes the production of epithelial-derived L-lactate in the small intestine and whether *V. cholerae* uses this metabolite *in vivo* to gain a fitness advantage during infection.

## RESULTS

### LldD is required for utilization of L-lactate by *Vibrio cholerae*

**Figure 1.**
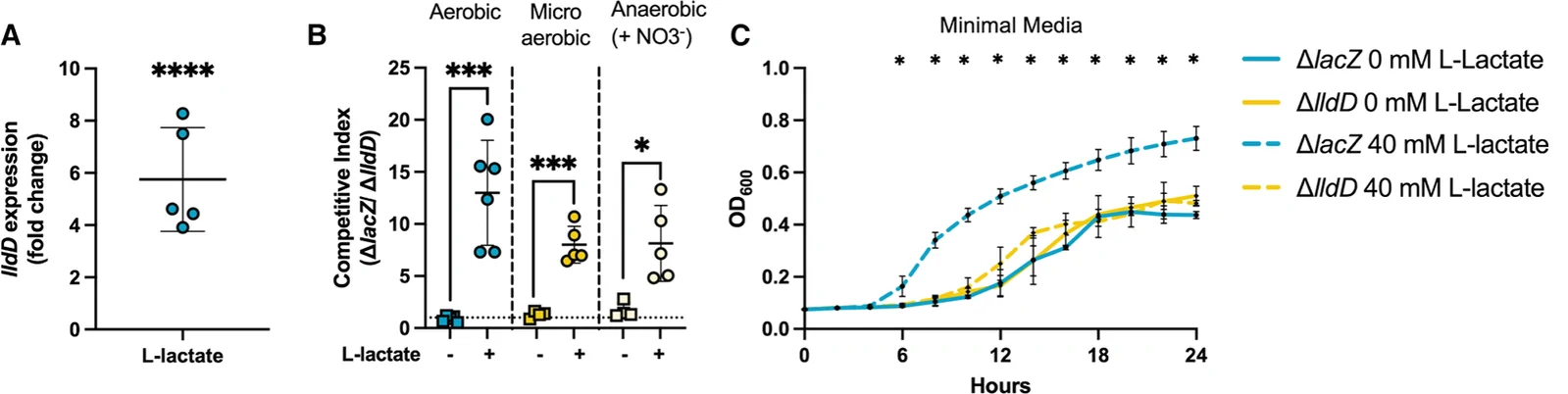
*V. cholerae* LldD is required for L-lactate utilization (A) Expression of *lldD* was measured on wild-type *V. cholerae* growing in M9 media 0.4% v/v glycerol in the presence or absence (control) of 40 mM L-lactate. Values represent fold change relative to control (*n* = 5). (B) Competitive growth assays *in vitro* were performed between a LldD-expressing strain (Δ*lacZ*) and a Δ*lldD* strain in the presence or absence of 40 mM L-lactate. M9 0.4% v/v glycerol media was inoculated with a 1:1 mixture of Δ*lacZ* and Δ*lldD* and incubated for 16 h at 37°C in aerobic, microaerobic, and anaerobic conditions. The competitive index (CI) was calculated as the ratio of Δ*lacZ*/Δ*lldD* recovered compared to the ratio of the inoculum (*n* = 4–6). (C) Growth curve of *V. cholerae* Δ*lacZ* (blue) and Δ*lldD* (yellow) in M9 0.4% v/v glycerol media (full line) or M9 0.4% v/v glycerol supplemented with 40 mM L-lactate (dotted line) grown for 24 h at 37°C (*n* = 4). Data are represented as mean ± SD. Each dot represents one biological replicate. For statistical analysis, unpaired *t* tests against same condition were performed. *p < 0.05; ***p < 0.001; ****p < 0.0001.

We have previously observed that during infection, L-lactate accumulates in the lumen of the small intestine, and *V. cholerae* upregulates the expression of *lldD*, the gene encoding the L-lactate dehydrogenase in a CT-dependent manner.^2^ To determine whether L-lactate alone can induce *lldD* expression, we cultured *V. cholerae* in the presence of L-lactate and found a significant increase in *lldD* expression (*p* < 0.05; Figure 1A), supporting the idea that *in vivo* induction of *lldD* during CT-induced disease reflects the availability of L-lactate in the intestinal lumen. Next, to investigate the role of L-lactate dehydrogenase in *V. cholerae* growth, we compared the fitness of the *V. cholerae* wild-type (using Δ*lacZ* for blue-white screening) and Δ*lldD* strains during growth with L-lactate. The *V. cholerae* wild-type strain exhibited a significant (*p* < 0.001) fitness advantage over the *ΔlldD* mutant under aerobic conditions (Figure 1B), consistent with a role for LldD in aerobic L-lactate metabolism. A similar significant fitness advantage was observed under microaerobic (*p* < 0.001) and anaerobic (*p* < 0.05) conditions in the presence of nitrate (Figure 1B), indicating that LldD supports growth across a range of physiologically relevant oxygen levels and alternative electron acceptors.

In agreement with the competition results, the Δ*lldD* mutant showed significantly (*p* < 0.05) impaired growth compared to the *V. cholerae* wild-type strain when grown in the presence of L-lactate (Figure 1C) but not in nutrient-rich media (Figure S1A). Importantly, LldD also conferred a growth advantage to *V. cholerae* strains incapable of producing CT (i.e., Δ*ctxAB* mutant background), confirming that CT production does not impact L-lactate utilization (Figures S1B and S1C). Finally, introduction of the cloned *lldD* gene on a plasmid (pMPG3) into the *lldD* mutant restored the growth defect in the presence of L-lactate (Figure S1D). Collectively, these findings demonstrate that LldD is required for efficient utilization of L-lactate by *V. cholerae* and confers a fitness advantage under diverse environmental conditions that are likely encountered in the gut environment where oxygen levels are low and nitrate becomes available.^6^

### L-lactate supports the luminal growth of *V. cholerae* in the small intestine

**Figure 2.**
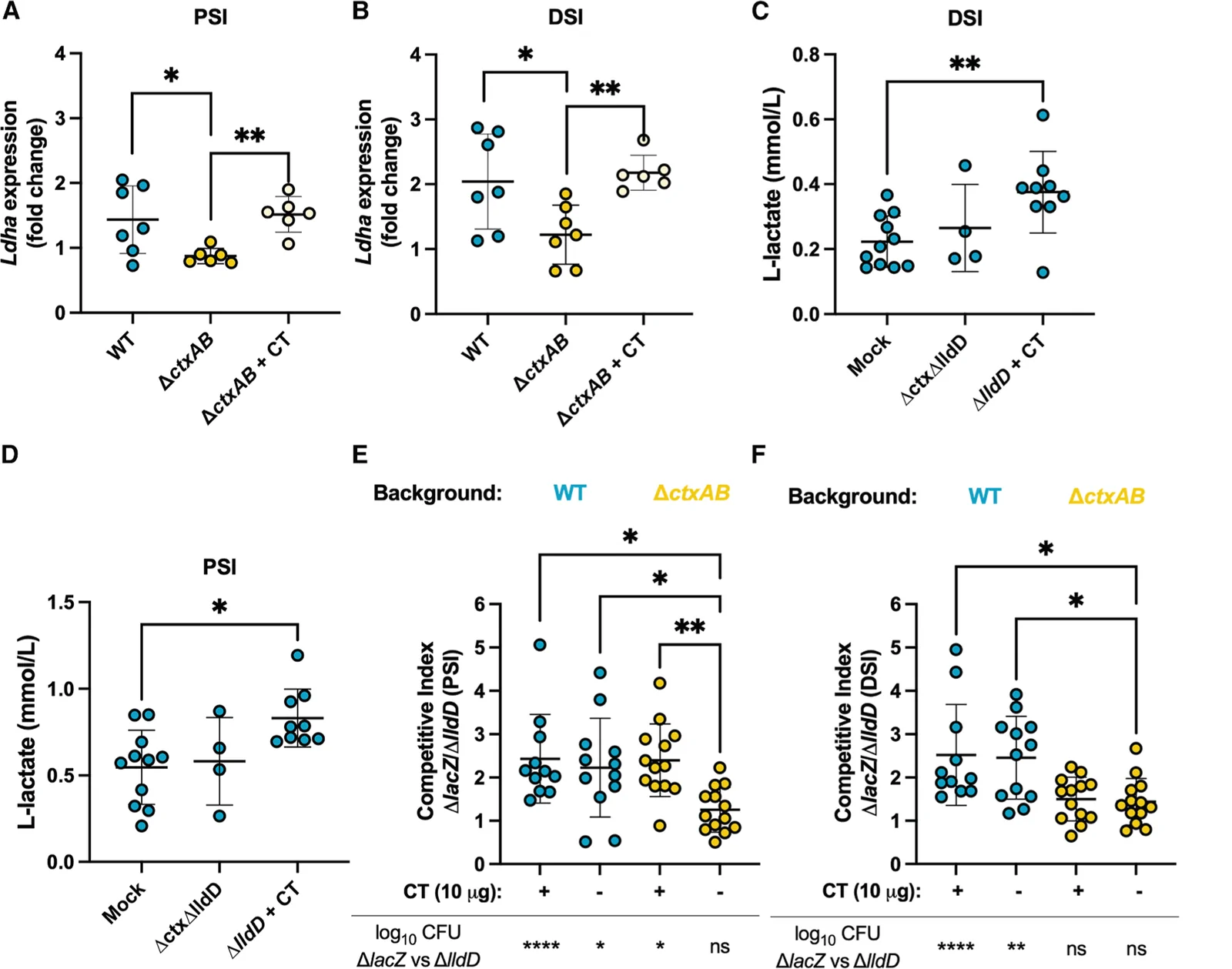
CT-dependent L-lactate utilization confers a fitness advantage to *V. cholerae* in the small intestine Expression of host L-lactate dehydrogenase *Ldha* was measured in the PSI (A) and DSI (B) of 7-day-old mice infected with wild-type (WT), Δ*ctxAB*, or Δ*ctxAB* co-inoculated with CT (10 μg). Mock-infected mice were used as control. Values represent fold change relative to mock-infected control (*n* = 6–7). Luminal L-lactate concentrations were quantified by GC-MS in the distal (C) and proximal (D) small intestine of mock-infected mice or mice infected with *ΔctxAB ΔlldD*, or Δ*lldD* co-inoculated with CT (n = 4–11). For (A–D), statistical significance was assessed using a one-way ANOVA followed by a Tukey’s multiple comparison test. Competitive assays in the suckling mouse model were performed using a LldD-expressing strain (Δ*lacZ*) and a Δ*lldD* strain. Seven-day-old pups were infected with a 1:1 mixture of Δ*lacZ* and Δ*lldD* with same genetic background: WT (blue) or Δ*ctxAB* (yellow). Bacteria recovered 22 h after infection from the PSI (E) and DSI (F) were used to calculate CI. When indicated, inoculums were supplemented with purified CT (10 μg) (*n* = 11–13). Data are represented as mean ± SD. Each dot represents data from one animal. For statistical analysis, a Kruskal-Wallis test followed by a Dunn’s multiple comparison test was performed. Paired *t* test were performed when comparing log_10_ CFU. *p < 0.05; **p < 0.01; ****p < 0.0001; ns, not statistically significant.

Since mammalian cells only generate the L-isomer of lactate through the activity of LDHA and we had previously found that CT-induced disease leads to elevated levels of L-lactate in the small intestinal lumen of infant rabbits,^2^ we next asked whether CT-induced disease has the potential to influence the availability of host-derived L-lactate through the direct upregulation of the mammalian L-lactate dehydrogenase gene (*Ldha*). To this end, expression of the host *Ldha* was quantified in the small intestine. *Ldha* expression was significantly elevated (*p* < 0.05) in both the distal and proximal small intestine in animals infected with wild-type *V. cholerae* but remained low in animals infected with the Δ*ctxAB* strain (Figures 2A and 2B). Furthermore, Δ*ctxAB*-infected mice that had been simultaneously treated orally with purified CT had significantly (*p* < 0.01) elevated *Ldha* levels similar to those observed in wild-type-infected mice (Figures 2A and 2B), demonstrating that CT is required to induce expression of host *Ldha*.

Our data suggested that use of L-lactate confers a fitness advantage to *V. cholerae* and that CT-induced disease leads to the upregulation of mammalian *Ldha*, which would be predicted to lead to elevated L-lactate production in the lumen. We therefore quantified luminal L-lactate in the small intestine by gas chromatography-mass spectrometry (GC-MS). Metabolomic analysis in neonatal mice is challenging because only small volumes can be recovered from the gut lumen, a limitation that is compounded when the small intestine is subdivided into proximal and distal compartments and when luminal metabolites may be depleted by bacterial consumption. Consistent with this, previous studies have found that detection of limiting luminal nutrients can be improved by infecting with a mutant unable to utilize the metabolite of interest.^7^ Accordingly, 7-day-old suckling mice were mock-infected or infected with the Δ*ctxAB* mutant, and a third group was infected with Δ*lldD* with concurrent exogenous CT treatment to maximize toxin exposure while limiting bacterial L-lactate oxidation. Luminal L-lactate concentrations were significantly elevated in both the distal (*p* < 0.01) and proximal (*p* < 0.05) small intestine in animals infected with the Δ*lldD* mutant concurrent with exogenous CT treatment but remained low in mock-infected animals or animals infected with the Δ*ctxAB* strain (Figures 2C and 2D). These findings were consistent with prior observations of a CT-dependent increase in L-lactate in infant rabbits infected with *V. cholerae*.^2^

To assess whether L-lactate supports the growth of *V. cholerae* in the lumen of the gastrointestinal tract during infection, 7-day-old suckling mice were infected with a 1:1 mixture of the *V. cholerae* wild-type and the Δ*lldD* mutant (CT-producing strains; left side, blue dots). After 22 h of infection, the wild-type *V. cholerae* significantly outcompeted the Δ*lldD* mutant in the lumen of the proximal (*p* < 0.05) and distal (*p* < 0.01) small intestine, indicating that use of L-lactate contributes to *V. cholerae* growth *in vivo* (Figures 2E and 2F).

Since CT-induced disease leads to increased L-lactate concentrations in the lumen of the small intestine and upregulation of L-lactate utilization genes in the pathogen,^2^ we next asked whether this L-lactate-dependent fitness advantage required CT-induced disease. To test this, we repeated the competitive infection using a Δ*ctxAB* mutant background that is unable to produce CT (right side, yellow dots). Consistent with the hypothesis that CT is required for L-lactate production, the competitive advantage conferred by LldD was significantly reduced (*p* < 0.05) in the absence of CT, and no fitness advantage was observed between Δ*ctxAB* and Δ*ctxAB ΔlldD* strains, suggesting that L-lactate-dependent growth requires CT-induced disease (Figures 2E and 2F). In wild-type *V. cholerae*-infected mice, concurrent exogenous CT treatment did not significantly increase the competitive advantage of the Δ*lacZ* strain over the Δ*lldD* mutant, suggesting that CT produced during wild-type infection is sufficient to drive L-lactate-mediated growth. Importantly, infection with the Δ*ctxAB* and Δ*ctxAB ΔlldD* strains concurrently with oral CT treatment led to a significant (*p* < 0.05) fitness advantage for the Δ*ctxAB* mutant in the proximal but not the distal small intestine, further supporting that CT-induced disease is responsible for L-lactate availability to enable pathogen growth in the small intestine (Figure 2E). This compartment-specific effect is consistent with our GC-MS measurements, showing higher luminal L-lactate in the proximal than the distal small intestine (Figures 2C and 2D).

Together, these findings demonstrate that CT promotes the generation of host-derived L-lactate in the small intestine and that *V. cholerae* benefits from the availability of this metabolite through LldD-dependent utilization of L-lactate during infection.

### Small intestinal epithelial cells are the primary source of L-lactate during CT-induced disease

**Figure 3.**
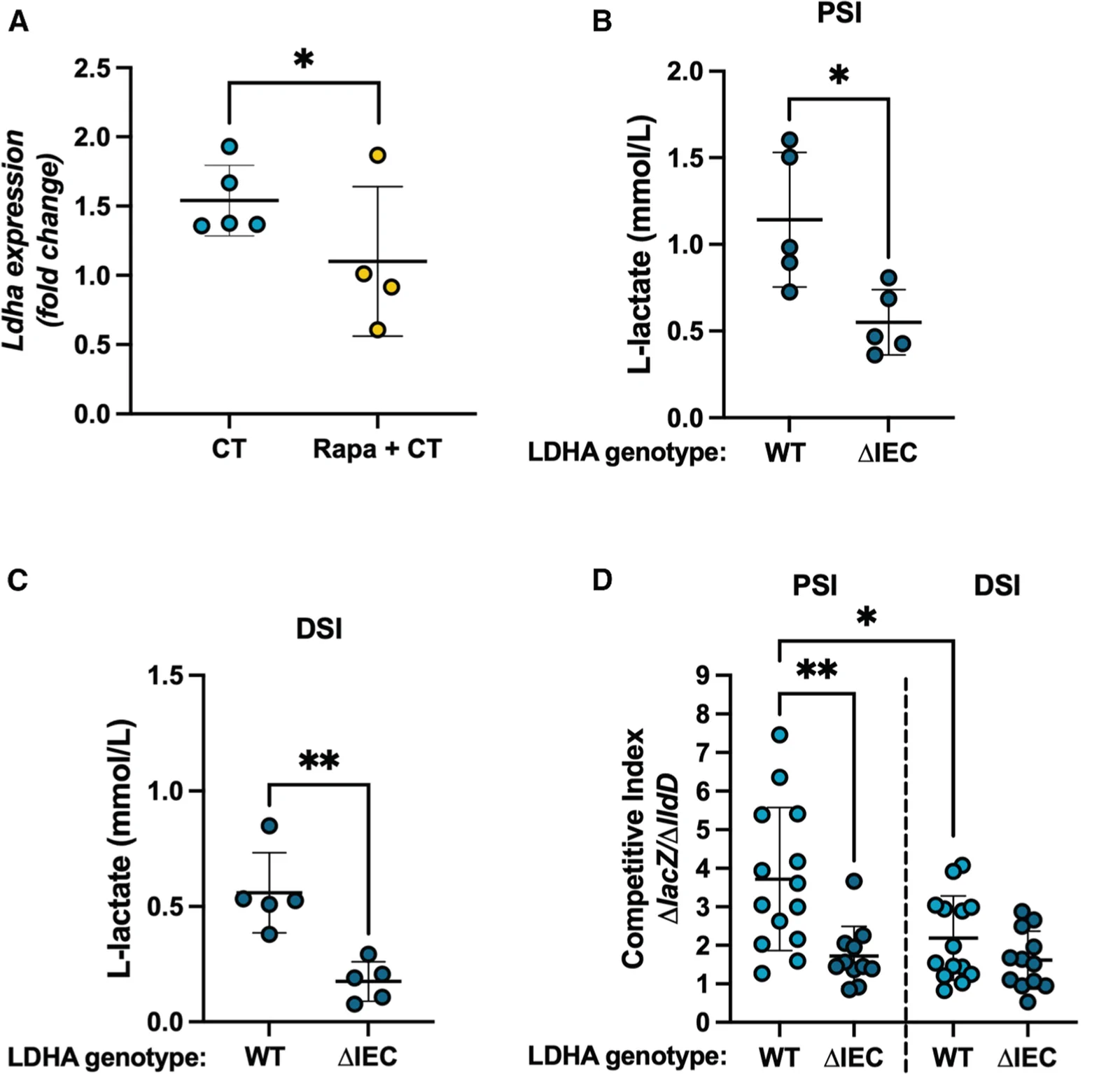
Epithelial-cell-derived L-lactate supports *V. cholerae* growth in the small intestine (A) Expression of L-lactate dehydrogenase *Ldha* was measured in IEC6 cells 2 h post-CT (10 μg) treatment. Where indicated, cells were pre-treated with 200 nM rapamycin (rapa) for 18 h before the addition of CT. For both treatments, media was used as control (*n* = 4–5). Quantification of L-lactate in proximal (B) and distal (C) small intestine contents of Ldha^ΔIEC^ and littermates (*n* = 5). (D) Competitive assays between an LldD-expressing strain (Δ*lacZ*) and a Δ*lldD* strain were conducted in 7-day-old Ldha^ΔIEC^ mice and wild-type (WT) littermates (*n* = 11–14). Data are represented as mean ± SD. Each dot represents data from one animal or one well. Unpaired *t* tests were performed for statistical analysis. *p < 0.05; **p < 0.01.

The intestinal epithelium is the primary target of CT,^8^ which acts on epithelial cells in the small intestine to drive ion secretion and water loss during infection.^1^ We next asked whether small intestinal epithelial cells are also the source of the L-lactate produced during CT-induced disease. Treatment of IEC-6 cells, a rat small intestinal epithelial cell line, with purified CT led to a significant (*p* < 0.05) increase in *Ldha* expression, supporting a role for small intestinal epithelial cells in L-lactate production during infection (Figure 3A). *Ldha* induction in IEC-6 cells was significantly (*p* < 0.05) abrogated by inhibition of mTORC1 signaling with rapamycin, consistent with a model in which CT stimulates *Ldha* expression via mTORC1-dependent activation of HIF-1α.^9^

To examine whether epithelial-derived L-lactate contributes to the intestinal luminal L-lactate pool, we infected mice that lack expression of *Ldha* in the intestinal epithelium (*Ldha*^ΔIEC^ mice) and littermate controls (WT) with *V. cholerae* wild-type and the Δ*lldD* mutant. Luminal L-lactate concentrations were also significantly reduced in both the proximal (*p* < 0.05) and the distal (*p* < 0.01) small intestine of *Ldha*^ΔIEC^ mice compared to littermate controls during infection with *V. cholerae* (Figures 3B and 3C). As before, to minimize depletion of luminal L-lactate by bacterial oxidation, we quantified luminal L-lactate during infection with the Δ*lldD* mutant. We did not administer exogenous CT in this genetic background because concurrent CT treatment was not tolerated in these mice, for reasons that remain unclear.

Consistent with our previous observations that L-lactate metabolism supports luminal growth in the small intestine (Figures 2C and 2D), the fitness advantage of wild-type *V. cholerae* (Δ*lacZ*) over the Δ*lldD* mutant was significantly reduced in the proximal small intestine of *Ldha*^ΔIEC^ mice compared to wild-type littermate controls (Figures 3D and S2B), indicating that *V. cholerae* primarily relies on epithelial-derived L-lactate to support growth in the lumen of the proximal small intestine during infection. Further supporting this conclusion, *Ldha* transcript levels were reduced by over 90% (*p* < 0.0001) in both proximal and distal small intestines of *Ldha*^ΔIEC^ mice compared to littermate controls infected with wild-type *V. cholerae*, confirming that intestinal epithelial-specific deletion effectively abrogates CT-dependent *Ldha* induction during infection (Figure S2A).

These findings identify epithelial cells in the small intestine as the primary source of CT-induced L-lactate and demonstrate that this host-derived metabolite promotes *V. cholerae* growth during infection. Together, these results uncover a novel mechanism by which CT, a phage-derived bacterial toxin, reprograms host epithelial metabolism to generate a nutrient that *V. cholerae* has evolved to exploit for growth in the gut during disease. This work has broad implications for the function of other bacterial toxins and advances our understanding of how toxin-mediated diarrheal disease confers a fitness advantage to enteric pathogens.

## DISCUSSION

Many pathogenic bacteria secrete toxins that elevate intracellular cyclic AMP (cAMP) or other cyclic nucleotides, leading to widespread disruption of host signaling and tissue function. While the disease-causing effects of these toxins have been well studied, recent work has begun to reveal how toxins can also reshape host metabolism to promote bacterial growth. Although CT was first described over six decades ago,^10^ the mechanisms by which it enhances *V. cholerae* fitness during infection have remained unclear.

Here, we identify a novel function for CT in the remodeling of host epithelial metabolism to generate a nutrient that directly fuels pathogen growth. We show that CT induces the expression of LDHA in epithelial cells of the small intestine, leading to increased availability of L-lactate in the lumen of the small intestine. *V. cholerae* exploits this metabolite using its own L-lactate dehydrogenase (LldD), which enables efficient use of L-lactate as carbon source and respiratory electron donor in the small intestine during disease. To our knowledge, CT is the first virulence factor identified to increase luminal L-lactate during infection, as no such single factor has been identified in infections caused by other pathogens.

Mammalian cells exclusively generate the L-isomer of lactate via LDHA, while gut microbes can produce both L- and D-lactate. *V. cholerae* genetically encodes the ability to import L-lactate via LldP and oxidize it using LldD. While *lldP* and *lldD* are strongly induced in the presence of CT during infection, their functional roles *in vivo* had not been previously defined. Here, we demonstrate that LldD confers a CT-dependent fitness advantage during infection and that this advantage is significantly diminished in the small intestine of mice lacking LDHA specifically in intestinal epithelial cells. Notably, this phenotype is compartment-specific. Consistent with this, our GC-MS measurements demonstrate that luminal L-lactate concentrations are higher in the proximal than the distal small intestine (Figures 2C and 2D). The basis for this proximal-to-distal gradient is unclear but could reflect the oral route and kinetics of CT exposure and/or region-specific lactate production and consumption by host and microbes, which together may impose stronger L-lactate limitation distally and thereby blunt LldD-dependent fitness benefits. These findings establish epithelial cells in the small intestine—the site where the pathogen colonizes and produces the toxin—as a key source of L-lactate during CT-induced disease. Notably, the modest residual advantage of wild-type *V. cholerae* in LDHA-deficient mice suggests that additional sources of L-lactate—potentially from non-epithelial cells such as phagocytes—may also contribute to pathogen growth.

In addition to serving as a nutrient, lactate can modulate host immune and barrier responses through signaling and post-translational modifications such as protein/histone lactylation,^11^ which likely influence bacterial colonization. Consistent with this, the absolute colony-forming unit (CFU) plots (Figure S2) show that bacterial burdens are not uniformly reduced in *Ldha*^ΔIEC^ mice and were trending higher in the distal small intestine, despite the reduced competitive advantage. Thus, while our *in vitro* data and competition experiments support a direct LldD-dependent growth advantage conferred by L-lactate, epithelial *Ldha* deletion *in vivo* appears to confound absolute-burden readouts *in vivo* by possibly altering immune responses during infection. Future studies will investigate the role of L-lactate and “lactylation” on colonization of *V. cholerae*.

The ability of enteric pathogens such as *Salmonella* Typhimurium to manipulate the gut environment and reach high luminal densities is essential for fecal-oral transmission.^12^ Our findings support the emerging paradigm that diarrheal disease functions not merely as a pathological consequence of infection, but as an evolved strategy to enhance pathogen growth and transmission. Our data suggest that CT-induced disease leads to an increase in the bioavailability of specific host-derived nutrients in the otherwise nutrient-scarce environment of the intestinal lumen. The distinct nature of these metabolites—serving as iron source, electron donor, and carbon source—indicates that the toxin activity leads to changes across a wide array of metabolic pathways rather than impacting a single one. As a result, *V. cholerae* likely has evolved to exploit these new available metabolites in order to reach high bacterial numbers in the intestinal lumen during disease. As CT also promotes massive fluid loss in humans, the resulting “rice water stool” can contain more than 10^11^ *V. cholerae* per liter. Notably, a previous proteomic analysis of stool from human cholera patients identified LldD as one of the most abundant *V. cholerae* proteins detected in stool samples,^13^ supporting the idea that L-lactate utilization may also be important for *V. cholerae* growth in the human gut during disease. These results uncover how CT-induced changes in host metabolism promote pathogen expansion in the gut, offering new insight into how toxin activity may contribute to fecal-oral transmission.

More broadly, this work reveals a direct, evolutionarily selected link between a horizontally acquired phage-encoded toxin, host metabolism, and bacterial nutrient acquisition. It raises the possibility that other bacterial toxins may operate through similar metabolic mechanisms. For example, pertussis toxin, which shares structural and enzymatic similarities with CT, may reprogram host metabolism in analogous ways during *Bordetella pertussis* infection. In sum, these findings expand our understanding of how microbial toxins contribute to pathogen ecology and evolution by modulating host physiology to promote pathogen growth and transmission.

### Limitations of the study

It is important to recognize the limitations of animal models of disease, particularly when studying a human-specific pathogen such as *V. cholerae*. This study defines a CT-dependent metabolic pathway in which epithelial LDHA drives luminal L-lactate production that is utilized by *V. cholerae* to promote pathogen growth during infection in a neonatal mouse model of experimental cholera. Because our *in vivo* experiments were performed in this neonatal mouse model, it remains to be determined how these findings translate to human infection. In addition, although LldD was previously identified as an abundant *V. cholerae* protein in human stool^13^ and we show here that LldD confers a fitness advantage in the small intestine of suckling mice, further studies will be needed to determine whether *V. cholerae* uses L-lactate during human infection. Furthermore, although our data support intestinal epithelial cells as a major source of luminal L-lactate during infection, we cannot exclude contributions from additional host or microbe-derived sources of L-lactate or from other metabolites that influence bacterial fitness during infection. Finally, although we demonstrate that epithelial-derived L-lactate contributes to pathogen growth in our model, the broader effects of CT-induced metabolic remodeling on the intestinal environment and host responses remain to be defined. Together, these findings establish L-lactate as a toxin-linked nutrient during infection, but further studies will be needed to define how this pathway operates in more complex and human-relevant settings.

## RESOURCE AVAILABILITY

### Lead contact

Further information and requests for resources and reagents should be directed to and will be fulfilled by the lead contact, Fabian Rivera-Chávez (fcrivera@health.ucsd.edu).

### Materials availability

Bacterial strains and plasmids generated in this study are available from the lead contact upon reasonable request.

### Data and code availability

- All numerical source data underlying the figures are provided with the published article and its supplemental information.
- This paper does not report original code.
- Any additional information required to reanalyze the data reported in this paper is available from the lead contact upon request.

## STAR★METHODS

### EXPERIMENTAL MODEL AND STUDY PARTICIPANT DETAILS

#### Bacterial strains and growth conditions

*V. cholerae* and *Escherichia coli* strains used in this study are listed in Table S1. *V. cholerae* and *E. coli* were routinely grown aerobically at 37°C in LB broth or on LB plates. When indicated, media was supplemented with streptomycin 100 μg/mL, carbenicillin 100 μg/mL, chloramphenicol 5 μg/mL (*V. cholerae*), chloramphenicol 20 μg/mL (*E. coli*) or X-gal. For infections, *V. cholerae* was grown in LB broth in the absence of antibiotics for 16 h.

For RNA extraction, wild-type *V. cholerae* was grown aerobically for 20 h in M9 media supplemented with 0.4% v/v glycerol and streptomycin in the presence or absence of 40 mM sodium L-lactate. After the incubation, bacteria were pelleted, resuspended in 1 mL of TRIzol reagent (Invitrogen, cat# 15596018) and stored at −80°C until used.

#### Mouse experiments

Wild-type C57BL/6 mice, *Ldha*^ΔIEC^ mice (*Ldha*^fl/fL^ Villin-Cre^+^) and littermates (*Ldha*^fl/fL^ Vil-Cre^−^) were generated using B6(Cg)-*Ldha*^*tm1c(EUCOMM)Wtsi*^*/*DatsJ (The Jackson Laboratory, stock #030112) and B6.Cg-Tg(*Vil1-cre*)1000Gum/J (The Jackson Laboratory, stock #021504) were used in this study. 7-day-old pups were separated from their dams 1 hour prior to infection. Pups were randomly allocated to experimental groups and orally gavaged with 1 × 10^6^ CFU *V. cholerae* alone or with 10 μg of CT in 50 μL of LB and placed inside a 30°C incubator. For competitive infections, *V. cholerae* strains were mixed in a 1:1 ratio and diluted to 1 × 10^6^ in 50 μL. Alternatively, pups were given LB as mock infection control. After 22 h, pups were euthanized, and the GI tracts were aseptically removed. The small intestine was cut into two pieces (proximal and distal), and the contents were squeezed and collected in 1 mL of PBS each. Tissues were snapfrozen in liquid nitrogen and stored at −80°C until used. Bacterial burden was determined by plating serial dilutions of the contents onto LB plates, supplemented with streptomycin. For competitive *in vivo* experiments, both inoculum and contents were plated onto plates containing streptomycin and X-gal. Competitive indices were calculated as (Output Δ*lacZ*/Output Δ*lldD*)/ (Input Δ*lacZ*/Input Δ*lldD*). Sex was not determined because experiments were performed in 7-day-old pups. Litters were randomly allocated to experimental groups and were expected to include both male and female pups.

All experiments in this study were approved by the Institutional Animal Care and Use Committee at the University of California San Diego.

#### Cell culture

IEC-6 cells were grown in Dulbecco’s Modified Eagle’s Medium (DMEM, Corning, cat# 10013CV) supplemented with 10% v/v fetal bovine serum (FBS) and 1% v/v penicillin-streptomycin in a 5% CO atmosphere at 37°C. Twelve-well plates were seeded with 2 × 10^5^ per well and the next day, media was replaced with fresh media alone or containing 200 nM rapamycin (Sigma-Aldrich, cat# 553210). After 18 h, CT was added to the wells in a final concentration of 100 μg/mL and the cells were incubated at 37°C for 2 h. After the incubation, media was removed and 1 mL of TRIzol reagent was added to each well to lyse the cells. Samples were stored at −80°C until RNA was extracted. IEC-6 cells were obtained from ATCC and were not further authenticated. Mycoplasma testing was not performed.

### METHOD DETAILS

#### Plasmids and strains construction

To generate a suicide plasmid for the clean deletion of *ctxAB*, a fragment containing two segments of homologous DNA flanking the genomic region of *ctxAB* (ctxABF1F2) was obtained by digesting pFRC1 with KpnI and XbaI (NEB). The released fragment was cloned into the plasmid pRE107 digested with the same enzymes. The resulting plasmid, pMPG1, was used for allelic replacement in *V. cholerae*.

Similarly, to generate a suicide plasmid for the clean deletion of *lldD* two segments of homologous DNA flanking the genomic region of *lldD* (lldDF1F2) were obtained by PCR and cloned into the plasmid pDS132 using Gibson Assembly. The resulting plasmid, pMPG2, was used for allelic replacement in *V. cholerae.*

The suicide plasmids were introduced into *V. cholerae* by conjugation with *E. coli* SM10. Transconjugants were selected on LB plates supplemented with streptomycin and carbenicillin (for integration of pMPG1) or streptomycin and chloramphenicol (for integration of pMPG2). Transconjugants were cultured overnight in LB (low sodium) in the absence of antibiotics and then plated onto LB with 15% w/v sucrose to select clones that had lost the plasmid. Mutants were confirmed by PCR using primers flanking the deleted region.

To construct the plasmid for complementation of *lldD*, the *lldD* gene was amplified together with 400 bp upstream, digested with KpnI and SacI and cloned into pWSK29. The resulting plasmid, pMPG3, as well as the empty vector pWSK29, were introduced into the *V. cholerae* Δ*lldD* mutant by electroporation. Transformants were selected on streptomycin and carbenicillin.

All primers used are listed on Table S2 and all strains in Table S3.

#### Growth curve *in vitro*

*V. cholerae* grown on an LB plate was used to inoculate an M9 liquid culture supplemented with 0.4% v/v glycerol and streptomycin and grown for 6 h on a shaker at 37°C. When necessary, the media was also supplemented with carbenicillin. The liquid culture was then diluted in M9 media to an OD_600_ of 0.22 and subsequently diluted 1/10^th^ in the same media with or without 40 mM sodium L-lactate (Sigma-Aldrich, cat# L7022). Two-hundred microliters of the resulting bacterial suspension were seeded in 96-well plate and incubated at 37°C with shaking in a plate reader SpectraMax ID5 (Molecular Devices) that measure OD_600_ every 30 min for a total of 24 h.

#### Competitive growth experiments *in vitro*

*V.* cholerae Δ*lacZ*, Δ*lldD*, Δ*ctxAB* Δ*lacZ* and Δ*ctxAB* Δ*lldD* grown on an LB culture overnight were diluted in M9 media supplemented with 0.4% v/v glycerol and streptomycin to an OD_600_ of 0.22 (equivalent to 2 × 10^8^ CFU/mL). The bacterial suspensions containing the Δ*lacZ* and the Δ*lldD* strains (or Δ*ctxAB* Δ*lacZ* and Δ*ctxAB* Δ*lldD*) were mixed in a 1:1 ratio and diluted to 2 × 10^6^ CFU/mL in the same media. For competitive growth experiments in aerobic and microaerobic condition, 5 mL of M9 0.4% v/v glycerol 100 μg/mL streptomycin with or without 40 mM L-lactate were inoculated with 50 μL of the inoculation mix (1 × 10^4^ CFUs). For experiments in anaerobic conditions cultures were additionally supplemented with 20 mM sodium nitrate. Cultures were incubated for 16 h at 37°C in a shaker (aerobic condition), in static with the lid completely closed (microaerobic condition) or in an anaerobic chamber (AnaeroPack, MGC) The inoculums as well as the cultures after incubation were serial diluted in PBS and plated in LB plates supplemented with streptomycin and X-gal. Competitive indices were calculated as: (Output Δ*lacZ*/Output Δ*lldD*)/(Input Δ*lacZ*/Input Δ*lldD*).

#### Measurement of L-lactate concentrations

Frozen samples were resuspended in 200 μL PBS, vortex homogenized and centrifuged at 6,000 g for 10 min. For each sample, 100 μL of supernatant was combined with 10 μL of a solution containing relevant concentration of internal standard. Succinic-2,2,3,3-d4 acid (CDN Isotopes D-0197) was used as the internal standard. Samples were dried without heat in a vacuum dryer.

The protocol for the separation and determination of lactic acid enantiomers by chiral derivatization was performed as previously described with modifications.^14^ Dried extracts were resolubilized in 100 μL of a 200 mg/mL solution of l-menthol (Sigma-Aldrich #W266523) in Toluene (Sigma-Aldrich #34866) followed by addition of 5 μL of acetyl chloride (Thermo Fisher Scientific #043262.AD). The mixture was incubated at 80°C for 2 h after which excess reagent was removed under a gentle flow of nitrogen gas. Afterward, 50 μL of acetyl chloride was added and the samples were incubated at room temperature for 1 h. Excess reagent was removed under a gentle flow of nitrogen gas. Finally, samples were dissolved in 100 μL of toluene and transferred to GCMS vials.

Samples were analyzed on an Agilent 8890 Gas Chromatograph coupled with an Agilent 7000D triple quadrupole mass spectrometer. One microliter of the sample was injected with a 1:20 split ratio at an injection temperature of 300°C on an HP 5ms Ultra Inert (2 × 15-m-length, 0.25 mm diameter, 0.25 μm film thickness) fused silica capillary column. Helium was used as the carrier gas with a constant flow of 1.2 mL/min. The GC oven temperature started at 60°C for 2 min, raised to 300°C at 17°C/min with a final hold for 2 min. The interface was heated to 300°C. The ion source was used in electron ionization (EI) mode. Analytes were quantified using selected ion monitoring (SIM) with a dwell time of 50 ms, with the monitored m/z detailed in the following table.

**Table:** 

Analyte	Quantifier ion (m/z)	Qualifiers (m/z)

L Lactate (Sigma-Aldrich #L7022)	83	123; 138; 87
D Lactate (Sigma-Aldrich #71716)	83	123; 138; 87
Succinic-2,2,3,3-d4 acid	123	105; 138

Efficient recovery of target metabolites was determined using deuterated compounds as internal standards. Quantification was based on external standards comprised of a series of dilutions of pure compounds, derivatized as described above at the same time as the samples. MassHunter Qualitative Analysis (version 10.0) and Agilent MassHunter Quantitative Analysis software (version 12.0) were used for data analysis.

#### RNA extraction and qRT-PCR

For RNA extraction, 200 μL of chloroform were added to bacterial or mammalian cells samples (resuspended in 1 mL of TRIzol reagent). Alternatively, snapfrozen proximal and distal small intestine samples were added to Green RINO RNA lysis kit (Next Advance) containing 1 mL of TRIzol reagent and homogenized for 5 min in Bullet Blender Storm Pro homogenizer (speed 10). The supernatants were added to new tubes containing 200 μL of chloroform. Samples with TRIzol reagent-chloroform were shaken vigorously and incubated for 15 min at room temperature. Samples were centrifuged at 12000 xg for 15 min at 4°C and the aqueous phase was transferred to a new tube. After the addition of 500 μL of 70% ethanol, the samples were transferred into a RNeasy micro column and continued isolation protocol according to manufacturer’s instructions. The concentration of the DNA-free RNA was quantified using Nanodrop One (Thermo Scientific). Samples were diluted to 10 ng/μL and stored at −80°C until used.

For qRT-PCR, SuperScript III Platinum One-Step qRT-PCR master mix kit (Invitrogen, cat# 12574026) was used following manufacturer’s instructions. Two technical replicate reactions with 2 μL of template RNA per sample per target gene were run on a CFX Opus 384 Real-time PCR thermocycler (Bio-Rad). Gene expression was normalized to a housekeeping gene (*actB* or *gyrB*) using the 2^−ΔΔCT^ method.

### QUANTIFICATION AND STATISTICAL ANALYSIS

#### Statistical analysis

Statistical analysis was performed using GraphPad Prism version 10. For comparison between two groups, an unpaired t-test was performed for parametric data and a Mann-Whitney test for non-parametric data. For comparison among three or more groups, a one-way ANOVA test followed by a Tukey’s multiple comparison test was performed for parametric data, and a Kruskal-Wallis test followed by a Dunn’s multiple comparisons test was performed for non-parametric data. Outliers were determined using the ROUT method (Q = 1).

## Supplementary Material

1

SUPPLEMENTAL INFORMATION

Supplemental information can be found online at https://doi.org/10.1016/j.celrep.2026.117509.

## Figures and Tables

**Table:** KEY RESOURCES TABLE

REAGENT or RESOURCE	SOURCE	IDENTIFIER

Bacterial and virus strains		

*Vibrio cholerae* C6706 (wild-type El Tor)	Lab Collection	N/A
*Vibrio cholerae* C6706 (*ΔlacZ*)	Ding, X et al^14^	N/A
*V. cholerae* C6706 (*ΔlldD*)	This paper	FRC178
*Vibrio cholerae* C6706 (*ΔctxAB*)	This paper	MPG-VC22
*Vibrio cholerae* C6706 (*ΔctxAB ΔlldD*)	This paper	MPG-VC20
*Vibrio cholerae* C6706 (*ΔctxAB ΔlacZ*)	This paper	MPG-VC29
*V. cholerae ΔlldD* carrying pWSK29	This paper	MPG-VC39
*V. cholerae ΔlldD* carrying pWSK29:*lldD*	This paper	MPG-VC44
*E. coli* SM10 carrying PDS132:ctxABF1F2	Rivera-Chávez et al^2^	FRC36
*E. coli* SM10 carrying *PRE107::ctxAB*F1F2	This paper	MPG-EC12
*E. coli* DH5α carrying PDS132:lldDF1F2	This paper	FRC160
*E. coli* CC118 carrying pWSK29:lldD	This paper	MPG-EC28

Chemicals, peptides, and recombinant proteins		

Sodium L-lactate	Sigma-Aldrich	Cat# L7022
D-lactate	Sigma-Aldrich	Cat# 71716
Rapamycin	Sigma-Aldrich	Cat# 553210
Cholera toxin	List Labs	Cat# 100 B
TRIzol reagent	Invitrogen	Cat# 15596018
l-menthol	Sigma-Aldrich	Cat# W266523
Toluene	Sigma-Aldrich	Cat# 34866
Acetyl chloride	Thermo Fisher	Cat# 043262.AD
Succinic-2,2,3,3-d4 acid	CDN Isotopes	Cat# D-0197

Critical commercial assays		

SuperScript III Platinum One-Step qRT-PCR Kit	Invitrogen	Cat# 12574026
RNeasy Mini Kit	QIAGEN	Cat# 74104
Green RINO RNA lysis kit	Next Advance	Cat# GREENR5-RNA

Experimental models: Cell lines		

Rat: IEC-6	ATCC	Cat# CRL-1592

Experimental models: Organisms/strains		

Mouse: C57BL/6	Jackson Laboratory	RRID: IMSR_JAX:000664
Mouse: B6(Cg)-Ldhatm1c(EUCOMM)Wtsi/DatsJ	Jackson Laboratory	RRID: IMSR_JAX:030112
Mouse: B6.Cg-Tg(Vil1-cre)1000Gum/J	Jackson Laboratory	RRID: IMSR_JAX:021504
Mouse: LdhaΔIEC: Ldhafl/fL Vil1-Cre+	Generated using B6(Cg)- Ldhatm1c(EUCOMM)Wtsi/DatsJ and B6.Cg-Tg(Vil1-cre)1000Gum/J	RRID: IMSR_JAX:030112; RRID: IMSR_JAX:021504
Mouse: littermate control: Ldhafl/fL Vil-Cre−	Generated using B6(Cg)- Ldhatm1c(EUCOMM)Wtsi/DatsJ and B6.Cg-Tg(Vil1-cre)1000Gum/J	RRID: IMSR_JAX:030112; RRID: IMSR_JAX:021504

Oligonucleotides		

Primers used in this study; see Table S2		

Recombinant DNA		

pWSK29	Wang et al.^15^	Cloning vector (CarbR)
PRE107	Miller et al.^16^	pGP704 modified suicide plasmid, sacB (CarbR)
PDS132	Philippe et al.^17^	pCVD442 modified suicide plasmid, sacB (CmR)
pMPG3	This paper	lldD ORF and 400 bp upstream, cloned into pWSK29 for complementation (CarbR)
pFRC1	Rivera-Chávez et al.2	pDS132 with regions upstream and downstream of ctxAB operon (CmR)
pMPG1	This paper	pRE107 with regions upstream and downstream of ctxAB operon (CarbR)
pMPG2	This paper	pDS132 with regions upstream and downstream of lldD ORF (CmR)

Software and algorithms		

GraphPad Prism	GraphPad Version 10.0 or higher	https://www.graphpad.com
NEBuilder assembly tool	New England Biolabs	https://nebuilder.neb.com
MassHunter Qualitative Analysis version 10.0	Agilent Technologies	https://www.agilent.com/
MassHunter Quantitative Analysis version 12.0	Agilent Technologies	https://www.agilent.com/
Geneious Prime (2026.1, 2026.0, 2025.2, 2025.1, 2025.0, and 2024.0)	Geneious	https://www.geneious.com

Other		

SpectraMax ID5 plate reader	Molecular Devices	N/A
CFX Opus 384 Real-Time PCR System	Bio-Rad	N/A
Bullet Blender Storm Pro homogenizer	Next Advance	N/A
Agilent 8890 Gas Chromatograph	Agilent Technologies	N/A
Agilent 7000D triple quadrupole mass spectrometer	Agilent Technologies	N/A

## Highlights

- LldD is required for L-lactate utilization by *Vibrio cholerae*
- Cholera toxin induces epithelial LDHA expression in the small intestine
- *V. cholerae* uses LldD to gain a CT-dependent fitness advantage *in vivo*
- Epithelial-derived L-lactate promotes *V. cholerae* fitness during infection

## References

[1] Faruque SM, Albert MJ, and Mekalanos JJ (1998). Epidemiology, genetics, and ecology of toxigenic *Vibrio cholerae*. Microbiol. Mol. Biol. Rev. 62, 1301–1314. 9841673 10.1128/mmbr.62.4.1301-1314.1998 PMC98947

[2] Rivera-Chávez F, and Mekalanos JJ (2019). Cholera toxin promotes pathogen acquisition of host-derived nutrients. Nature 572, 244–248. 31367037 10.1038/s41586-019-1453-3 PMC6727848

[3] Gillis CC, Hughes ER, Spiga L, Winter MG, Zhu W, Furtado de Carvalho T, Chanin RB, Behrendt CL, Hooper LV, Santos RL, and Winter SE (2018). Dysbiosis-associated change in host metabolism generates lactate to support *Salmonella* growth. Cell Host Microbe 23, 54–64.e6. 29276172 10.1016/j.chom.2017.11.006 PMC5764812

[4] Sinha R, LeVeque RM, Callahan SM, Chatterjee S, Stopnisek N, Kuipel M, Johnson JG, and DiRita VJ (2024). Gut metabolite L-lactate supports *Campylobacter jejuni* population expansion during acute infection. Proc. Natl. Acad. Sci. USA 121, e2316540120. 10.1073/pnas.2316540120 PMC10786315 38170751

[5] Taylor SJ, Winter MG, Gillis CC, Silva L.A.d., Dobbins AL, Muramatsu MK, Jimenez AG, Chanin RB, Spiga L, Llano EM, (2022). Colonocyte-derived lactate promotes *E. coli* fitness in the context of inflammation-associated gut microbiota dysbiosis. Microbiome 10, 200. 36434690 10.1186/s40168-022-01389-7 PMC9701030

[6] Bueno E, Sit B, Waldor MK, and Cava F (2018). Anaerobic nitrate reduction divergently governs population expansion of the enteropathogen *Vibrio cholerae*. Nat. Microbiol. 3, 1346–1353. 30275512 10.1038/s41564-018-0253-0 PMC6443258

[7] Winter SE, Thiennimitr P, Winter MG, Butler BP, Huseby DL, Crawford RW, Russell JM, Bevins CL, Adams LG, Tsolis RM, (2010). “Gut inflammation provides a respiratory electron acceptor for *Salmonella*”. Nature 467, 426–429. 20864996 10.1038/nature09415 PMC2946174

[8] Van Heyningen WE, Van Heyningen S, and King CA (1976). The nature and action of cholera toxin. Ciba Found. Symp. 42, 73–88. 10.1002/9780470720240.ch5 791600

[9] Kierans SJ, and Taylor CT (2021). Regulation of glycolysis by the hypoxia-inducible factor (HIF): implications for cellular physiology. J. Physiol. 599, 23–37. 33006160 10.1113/JP280572

[10] De SN (1959). Enterotoxicity of bacteria-free culture filtrate of *Vibrio cholerae*. Nature 183, 1533–1534. 13666809 10.1038/1831533a0

[11] Chen AN, Luo Y, Yang YH, Fu JT, Geng XM, Shi JP, and Yang J (2021). Lactylation, a Novel Metabolic Reprogramming Code: Current Status and Prospects. Front. Immunol. 12, 688910. 10.3389/fimmu.2021.688910 PMC8222712 34177945

[12] Lawley TD, Bouley DM, Hoy YE, Gerke C, Relman DA, and Monack DM (2008). Host transmission of *Salmonella enterica* serovar Typhimurium is controlled by virulence factors and indigenous intestinal microbiota. Infect. Immun. 76, 403–416. 17967858 10.1128/IAI.01189-07 PMC2223630

[13] LaRocque RC, Krastins B, Harris JB, Lebrun LM, Parker KC, Chase M, Ryan ET, Qadri F, Sarracino D, and Calderwood SB (2008). Proteomic analysis of *Vibrio cholerae* in human stool. Infect. Immun. 76, 4145–4151. 18591230 10.1128/IAI.00585-08 PMC2519452

[14] Ding X, Lin S, Weng H, and Liang J (2018). “Separation and determination of the enantiomers of lactic acid and 2-hydroxyglutaric acid by chiral derivatization combined with gas chromatography and mass spectrometry”. J. Separ. Sci. 41, 2576–2584. 10.1002/jssc.201701555 29603663

[15] Wang RF, and Kushner SR (1991). Construction of versatile low-copy-number vectors for cloning, sequencing and gene expression in Escherichia coli. Gene 100, 195. 2055470

[16] Miller VL, and Mekalanos JJ (1988). A novel suicide vector and its use in construction of insertion mutations: osmoregulation of outer membrane proteins and virulence determinants in Vibrio cholerae requires toxR. J. Bacteriol. 170, 2575–2583. 2836362 10.1128/jb.170.6.2575-2583.1988 PMC211174

[17] Philippe N, Alcaraz JP, Coursange E, Geiselmann J, and Schneider D (2004). Improvement of pCVD442, a suicide plasmid for gene allele exchange in bacteria. Plasmid 51, 246–255. 15109831 10.1016/j.plasmid.2004.02.003

