# Supplementary material for: Cholera-toxin-induced disease generates epithelial-cell-derived L-lactate that promotes Vibrio cholerae growth in the small intestine
Source: Cell Rep. Author manuscript; Available in PMC 2026 Aug 31. (PMC13528590; doi:10.1016/j.celrep.2026.117509)
Supplement: Supplementary file 1 [file NIHMS2190744-supplement-1.pdf]

Cell Reports, Volume *45*

Supplemental information

Cholera-toxin-induced disease generates epithelial-cell-derived L-lactate that promotes *Vibrio cholerae* growth in the small intestine

Maria de la Paz Gutierrez, Jenna L. Hall, Hugo L.P. Masson, Maria G. Winter, Sebastian E. Winter, and Fabian Rivera-Chávez

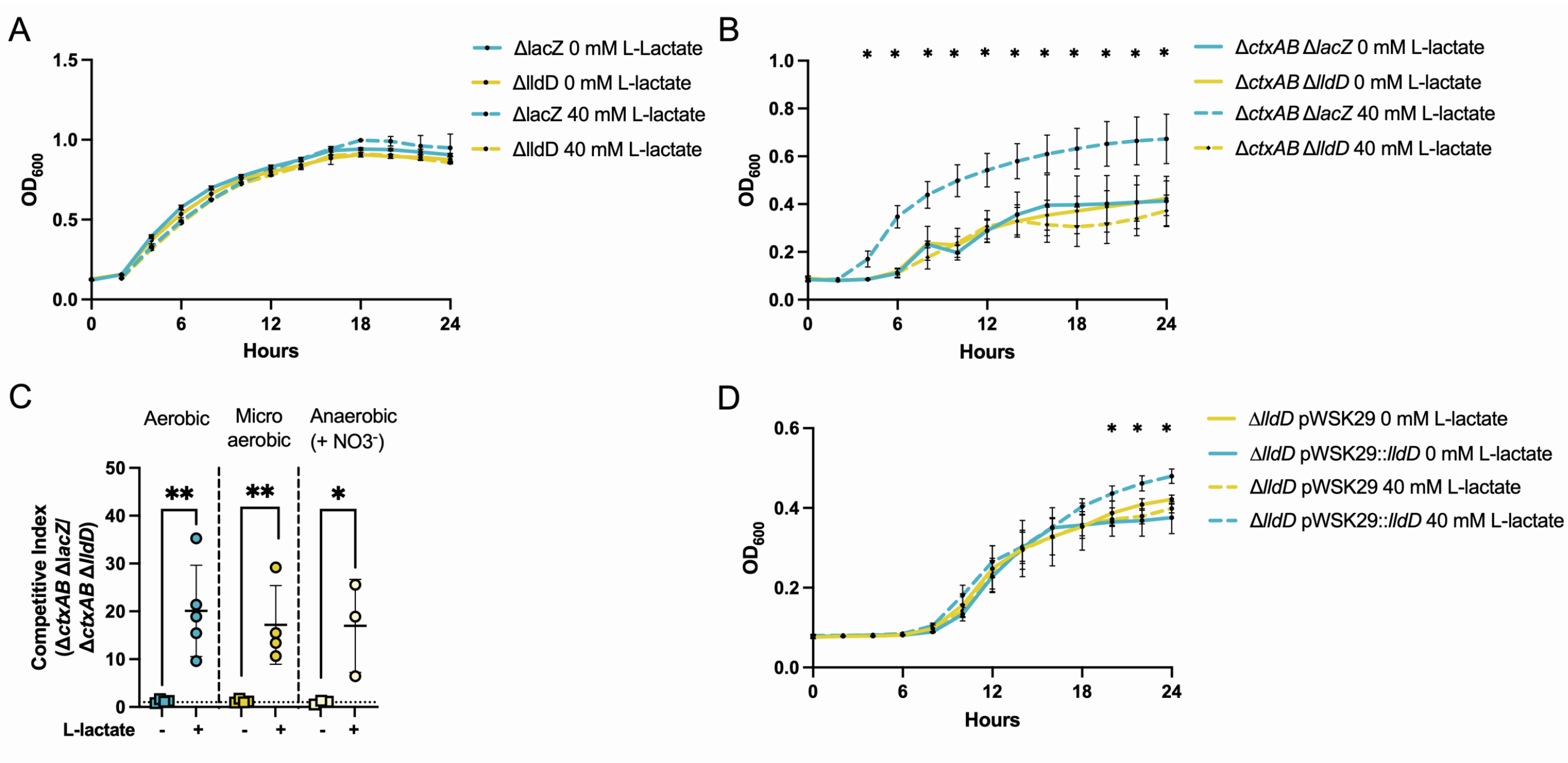

Figure S1. LldD supports *V. cholerae* growth *in vitro* in the presence of L-lactate

(A) Growth curve of *V. cholerae* Δ*lacZ* (blue) and Δ*lldD* (yellow) in LB broth in the presence (dotted line) or absence (full line) of 40 mM L-lactate. **(B)** Growth curve of *V. cholerae* Δ*ctxAB* Δ*lacZ* (blue) and Δ*ctxAB* Δ*lldD* (yellow) in LB broth in the presence (dotted line) or absence (full line) of 40 mM L-lactate. **(C)** Competitive growth assays *in vitro* were performed between Δ*ctxAB* Δ*lacZ* and Δ*ctxAB* Δ*lldD* in the presence or absence of 40 mM L-lactate. The competitive index (CI) was calculated as the ratio of Δ*ctxAB* Δ*lacZ*/Δ*ctxAB* Δ*lldD* recovered compared to the ratio of the inoculum (n= 3-5). **(D)** Growth curves of *V. cholerae* Δ*lldD* harboring the *lldD* complementation plasmid pMPG3 (pWSK29::*lldD*; blue) and Δ*lldD* harboring the empty vector (pWSK29; yellow) in M9 medium containing 0.4% v/v glycerol (solid lines) or M9 medium containing 0.4% v/v glycerol supplemented with 40 mM L-lactate (dotted lines) grown for 24 h at 37°C (n= 4). Data are represented as mean +/- SD. Each dot represents one biological replicate. For statistical analysis, unpaired t-tests against same condition were performed. * $p < 0.05$; ** $p < 0.01$.

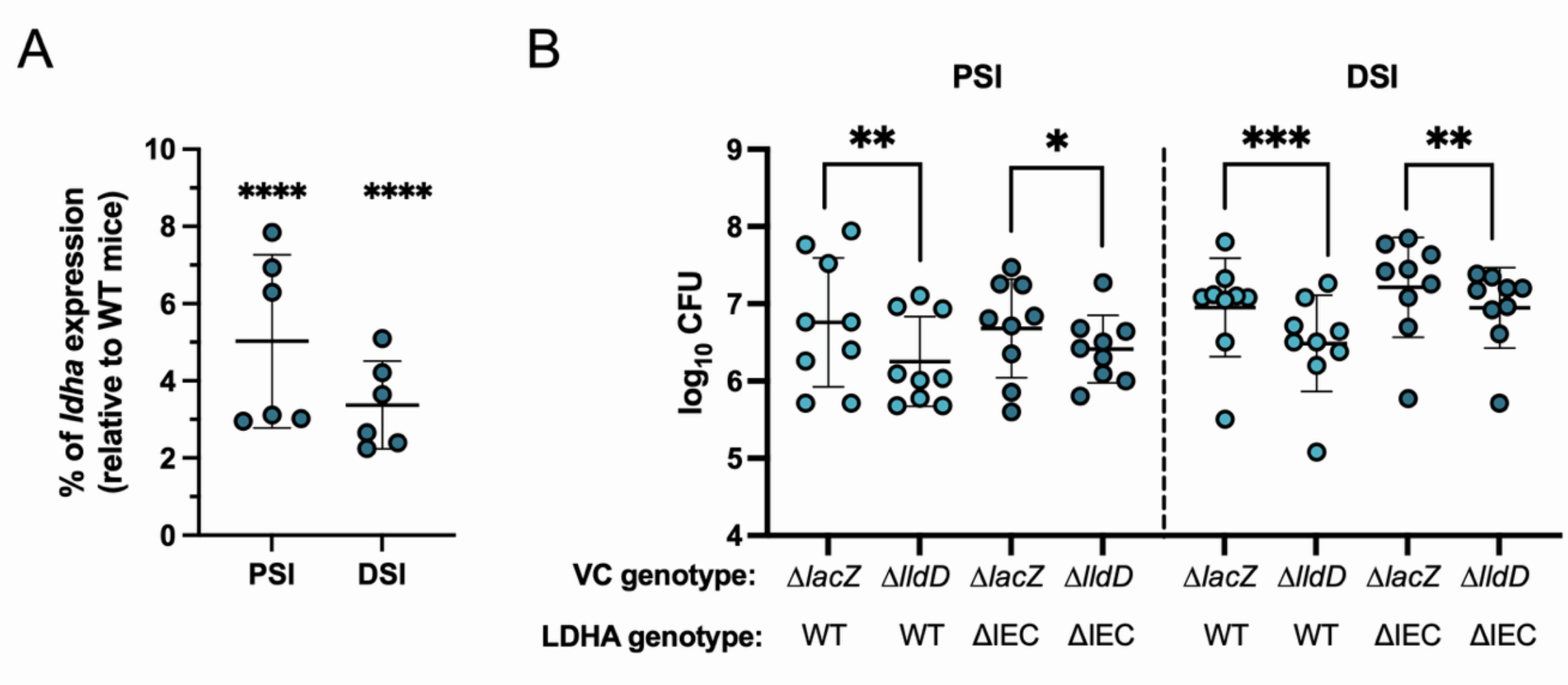

Figure S2. Epithelial *Ldha* deletion reduces *Ldha* expression during *V. cholerae* infection

(A) Expression of *Ldha* in Ldha$^{\Delta IEC}$ mice infected with *V. cholerae*, shown as percentage of *ldha* expression in WT mice infected with *V. cholerae* (n=6). **(B)** Total CFU of Δl*acZ* and Δ*lldD* recovered from proximal (PSI) and distal (DSI) small intestine in competitive infection experiments in Ldha$^{\Delta IEC}$ mice and littermates (WT) shown in Fig. 3D. Data are represented as mean +/- SD. Each dot represents data from one animal. Unpaired t-test were performed for statistical analysis * $p < 0.05$; ** $p < 0.01$; *** $p < 0.001$; **** $p < 0.0001$

Table S1. Bacterial strains used in this study

**Strain**	**Genotype**	**Reference**
C6706	*V. cholerae* Wild-type El tor (Sm^{R})	Lab collection
C6706 ΔlacZ	*V. cholerae ΔlacZ* (Sm^{R})	[S1]
FRC178	*V. cholerae ΔlldD* (Sm^{R})	This study
MPG-VC22	*V. cholerae ΔctxAB* (Sm^{R})	This study
MPG-VC20	*V. cholerae ΔctxAB ∆lldD* (Sm^{R})	This study
MPG-VC29	*V. cholerae ΔctxAB ∆lacZ* (Sm^{R})	This study
MPG-VC39	*V. cholerae ΔlldD* carrying pWSK29 (Sm^{R}, Carb^{R})	This study
MPG-VC44	*V. cholerae ΔlldD* carrying pWSK29::*lldD* (Sm^{R}, Carb^{R})	This study
FRC36	*E. coli* SM10 carrying PDS132::ctxABF1F2 (Cm^{R})	[2]
MPG-EC12	*E. coli* SM10 carrying *PRE107::ctxAB*F1F2 (Carb^{R})	This study
FRC160	*E. coli* DH5α carrying PDS132::lldDF1F2 (Carb^{R})	This study
MPG-EC28	*E. coli* CC118 carrying pWSK29::lldD (Carb^{R})	This study

Table S2. Primers used in this study

Name	Sequence	Reference
Ldha (M) Fw	TGTCTCCAGCAAAGACTACTGT	[S2]
Ldha (M) Rv	GACTGTACTTGACAATGTTGGGA	[S2]
ActB (M) Fw	GCTGAGAGGGAAATCGTGCGTG	[S3]
ActB (M) Rv	CCAGGGAGGAAGAGGATGCGG	[S3]
LdhA (R) Fw	GATCTCGCGCACGCTACTG	[S4]
LdhA (R) Rv	TTTGAATCTTTTGGGACCGCTT	[S4]
ActB (R) Fw	GCTCTATCCTGGCCTCACTGTCCA	This study
ActB (R) Rv	CCGGACTCATCGTACTCCTGCTTG	This study
ctx delA	GAGGTACCGCATGCGATATCGAGCTTTTACAGGG GGAAATGATGC	[2]
ctx delD	AATTTGTGGAATTCCCGGGAGAGCTCTAGACGGG GGTCTACCACA	[2]
lldD qF	TCTGGTTCCAGCTTTACGTTT	[2]
lldD qR	TATCCACGGTGAACACCAGA	[2]
gyrB Fw	GTGGTTTCGGTGAAAGTGCC	[2]
gyrB Rv	GTTTTTCGCTTCGCTTGGGT	[2]
lldD delA	GaggtaccgcatgcgatatcgagctGCCGGCTCTAACACAGT CTC	This study
lldD delB	attatcttttgacGGAAATGATCATAATGCAATCCTATT	This study
lldD delC	tatgatcatttccGTCAAAAGATAATAAAAGTATCCCCTA	This study

lldD delD	AatttgtggaattcccgggagagctGCCTTGGTTTTGCACTTCAT	This study
LldD Prm Fw	CATGGTACCCTCTAACACAGTCTCCAAC	This study
lldD STP Rv	GTAGAGCTCTTATCTTTTGACTAATGAATCG	This study

Table S3. Plasmids used in this study

**Name**	**Characteristics**	**Reference**
pWSK29	Cloning vector (Carb^R)	[S[5]]
PRE107	pGP704 modified suicide plasmid, *sacB* (Carb^R)	[S[6]]
PDS132	pCVD442 modified suicide plasmid, *sacB*, (Cm^R)	[S[7]]
pMPG3	*lldD* ORF and 400 bp upstream, cloned into pWSK29 for complementation (Carb^R)	This study
pFRC1	pDS132 with regions upstream and downstream of *ctxAB* operon (Cm^R)	[2]
pMPG1	PRE107 with regions upstream and downstream of *ctxAB* operon (Carb^R)	This study
pMPG2	pDS132 with regions upstream and downstream of *lldD* ORF (Cm^R)	This study

REFERENCES

1. Cameron, D.E., Urbach, J.M., and Mekalanos, J.J. (2008). A defined transposon mutant library and its use in identifying motility genes in *Vibrio cholerae*. *Proc. Natl. Acad. Sci. U.S.A.* **105**, 8736–8741.

2. Huang, X., Xie, X., Wang, H., Xiao, X., Yang, L., Tian, Z., Guo, X., Zhang, L., Tang, H., Xie, X., *et al.* (2017). PDL1 and LDHA act as ceRNAs in triple negative breast cancer by regulating miR-34a. *J. Exp. Clin. Cancer Res.* **36**, 129.

3. Becker, S., Oelschlaeger, T.A., Wullaert, A., Pasparakis, M., Wehkamp, J., Stange, E.F., and Gersemann, M. (2013). Bacteria regulate intestinal epithelial cell differentiation factors both *in vitro* and *in vivo*. *PLoS One* **8**, e55620.

4. Hernandez, A.H., Curi, R., and Salazar, L.A. (2015). Selection of reference genes for expression analyses in liver of rats with impaired glucose metabolism. *Int. J. Clin. Exp. Pathol.* **8**, 3946–3954.

5. Wang, R. F. and S. R. Kushner (1991). Construction of versatile low-copy-number vectors for cloning, sequencing and gene expression in Escherichia coli. *Gene* **100**: 195-199.

6. Miller, V. L. and J. J. Mekalanos (1988). A novel suicide vector and its use in construction of insertion mutations: osmoregulation of outer membrane proteins and virulence determinants in *Vibrio cholerae* requires toxR. *J Bacteriol* **170**(6): 2575-2583.

7. Philippe, N., J. P. Alcaraz, E. Coursange, J. Geiselmann and D. Schneider (2004). Improvement of pCVD442, a suicide plasmid for gene allele exchange in bacteria. *Plasmid* **51**(3): 246-255.s
